# Comparison of C2-3 Pedicle Screw Fixation With C2 Spinous Muscle Complex and Iliac Bone Graft for Instable Hangman Fracture

**DOI:** 10.3389/fsurg.2021.723078

**Published:** 2021-11-26

**Authors:** Dingli Xu, Kaifeng Gan, Yang Wang, Yulong Wang, Weihu Ma

**Affiliations:** ^1^The Affiliated Hospital of Medical School, Ningbo University, Ningbo, China; ^2^Ningbo City Medical Treatment Center Lihuili Hospital, Ningbo, China; ^3^Ningbo No.6 Hospital, Ningbo, China

**Keywords:** instable Hangman fracture, C2 spinous muscle complex, donor-site complications, surgical procedures, bone graft

## Abstract

**Purpose:** To compare the effect between C2 spinous muscle complex graft and iliac bone graft in C2-3 pedicle screw fixation for instable Hangman fracture. Using axial spinous muscle complex instead of iliac bone for instable Hangman fracture can decrease neck pain, bone donor site complication, and operation time.

**Method:** The outcomes of C2-3 pedicle screw fixation with C2 spinous muscle complex were compared with iliac bone graft in 18 and 21 patients with instable Hangman fracture. The mean age was 49.1 ± 15.8 years in the complex group and 55.3 ± 12.2 years in the Iliac group, and the mean time to surgery of the patients was 3.3 ± 0.6 days in the complex group and 3.6 ± 0.9 days in the iliac group. Outcome measures including operation time, blood loss, visual analog scale (VAS) for pain, Japanese orthopedic association score (JOA), American spine injure association classification (ASIA), and bone fusion time were collected from medical records. In addition, the postoperative complications were also recorded.

**Results:** There were significant differences in operation time and interoperative blood loss between the two groups (*P* < 0.01). Also a significant difference was found in VAS score and JOA score between the two groups (*P* = 0.0012 and *P* < 0.001, respectively) at 1-month follow-up, whereas, no significant difference was found at other visit time. In the final visit, all patients showed good bone fusion, and two patients shows incision edema and exudation in the iliac group.

**Conclusion:** C2-3 pedicle screw fixation with C2 spinous muscle complex graft maybe a feasible and safe procedure for instable Hangman fracture.

## Introduction

Hangman fracture, known as traumatic spondylolisthesis of the axis, was first discovered in 1866 in dead criminals from judicial hanging and was named by Schneider et al. in 1965 (1). According the Levine and Edwards classification (2), fractures with severe circumferential disco-ligamentous injuries and a variable degree of translation or angulation of the C2 on the C3 vertebra are thought to be unstable and require rigid immobilization. The commonly used surgical methods are posterior C2-3 pedicle screw fixation and iliac bone graft, which have shown good clinical outcomes (3). Biomechanical studies in cadavers have suggested that posterior C2-3 fusion is possibly better than other techniques such as anterior graft and plating and C2 par fixation (4). However, some researchers have reported neck pain and donor-site complications such as pain, hematoma, oedema, infection, and pseudoarthrosis (5).

Therefore, researchers have increased interest in the axial spinous process. Sinha and Goyal used the C2 spinous process as a bone graft and waged it using titanium cables for C1-2 posterior fusion in five patients with atlantoaxial dislocation to minimize the donor-site complications and posterior neck pain (6). Similarly, our group reported that 27 patients with atlantoaxial fracture who were treated with C1-2 pedicle screw fixation combined with axial spinous muscle complex had satisfactory recovery (7). Moreover, we used the axial spinous process in atlantoaxial surgery (8). Besides, many studies reported that the preservation of muscle attachments of cervical spine is beneficial in cervical ROM and axial symptom. Riew et al. reviewed 11 articles on preserving the C2 muscle attachments and/or C7-preserving cervical laminoplasty and reported a similar result that preservation of the posterior cervical muscle has better clinical outcomes (9). Preservation of the cervical muscles such as the semispinalis cervicis muscle prevents postoperative neck pain and maintains cervical alignment.

**Figure 1:**
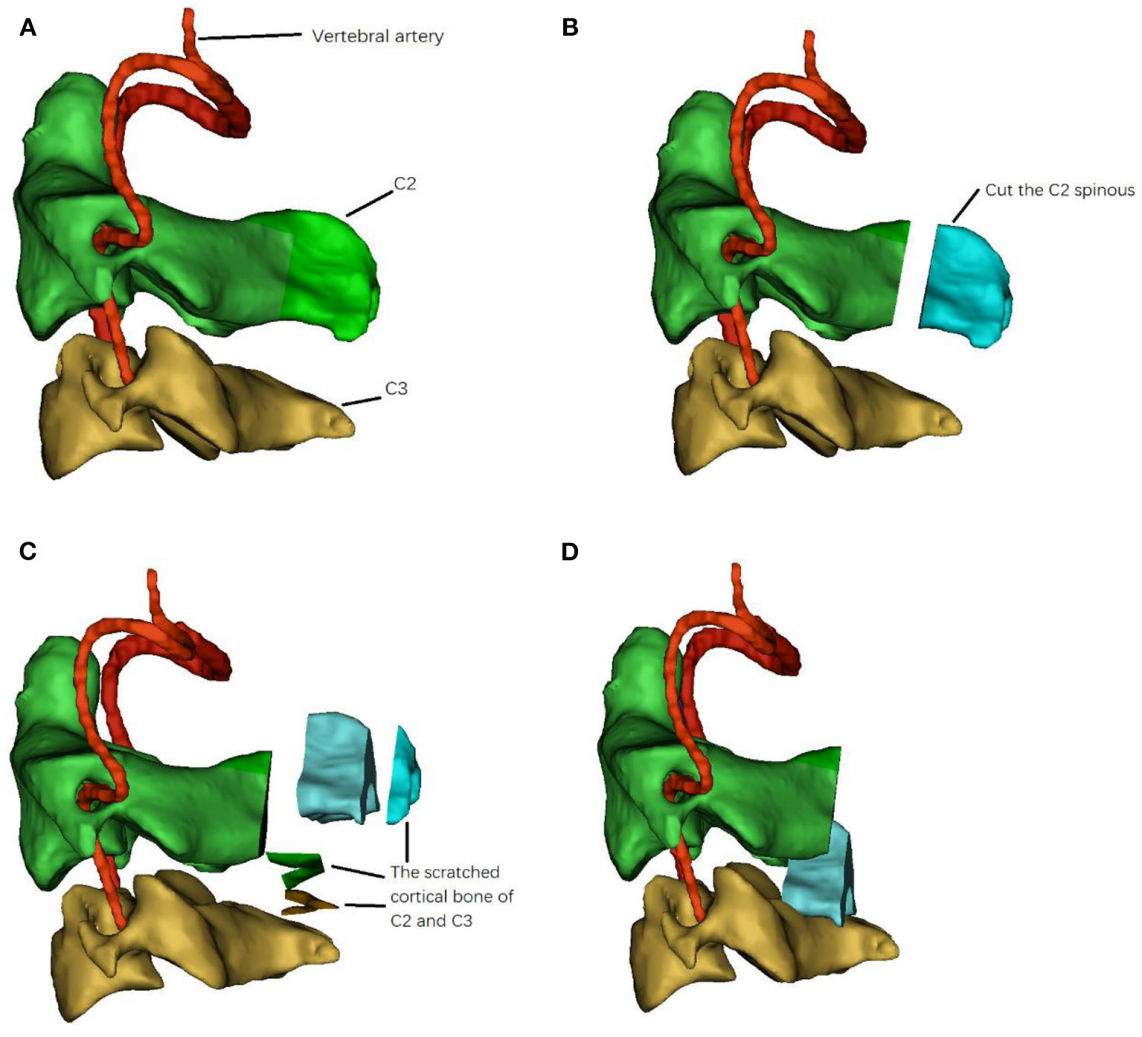
The diagram of C2 spinous muscle complex graft. **(A)** the anatomy of C2, C3 and vertebral artery, **(B)** Cut down the posterior 3/4 of C2 spinous, **(C)** scratch the cortical of C2 and C3 for bone graft, **(D)** displace the C2 spinous muscle complex graft into C2-3 posterior arch.

In this study, we performed posterior C2-3 pedicle screw fixation using the axial spinous muscle complex to minimize the posterior complications of unstable Hangman fractures, and the schematic of the C2 spinous muscle complex graft is shown in Figure 1. We compared the outcomes between pedicle screw fixation with axial spinous muscle complex and with iliac bone grafts for unstable Hangman fractures.

## Materials and Methods

In this study, we retrospectively reviewed patients with unstable Hangman fractures who were treated with C2-3 pedicle screw fixation with axial spinous muscle complex or iliac bone graft between September 2014 and April 2017 at Ningbo Number 6 Hospital. All procedures involving human participants were performed in accordance with the ethical standards of the institution and the 1964 Helsinki Declaration, and its later amendments or comparable ethical standards. The study was approved by the Bioethics Committee of the Ningbo No.6 Hospital of Ningbo University. All patients provided informed consent.

**Figure 2:**
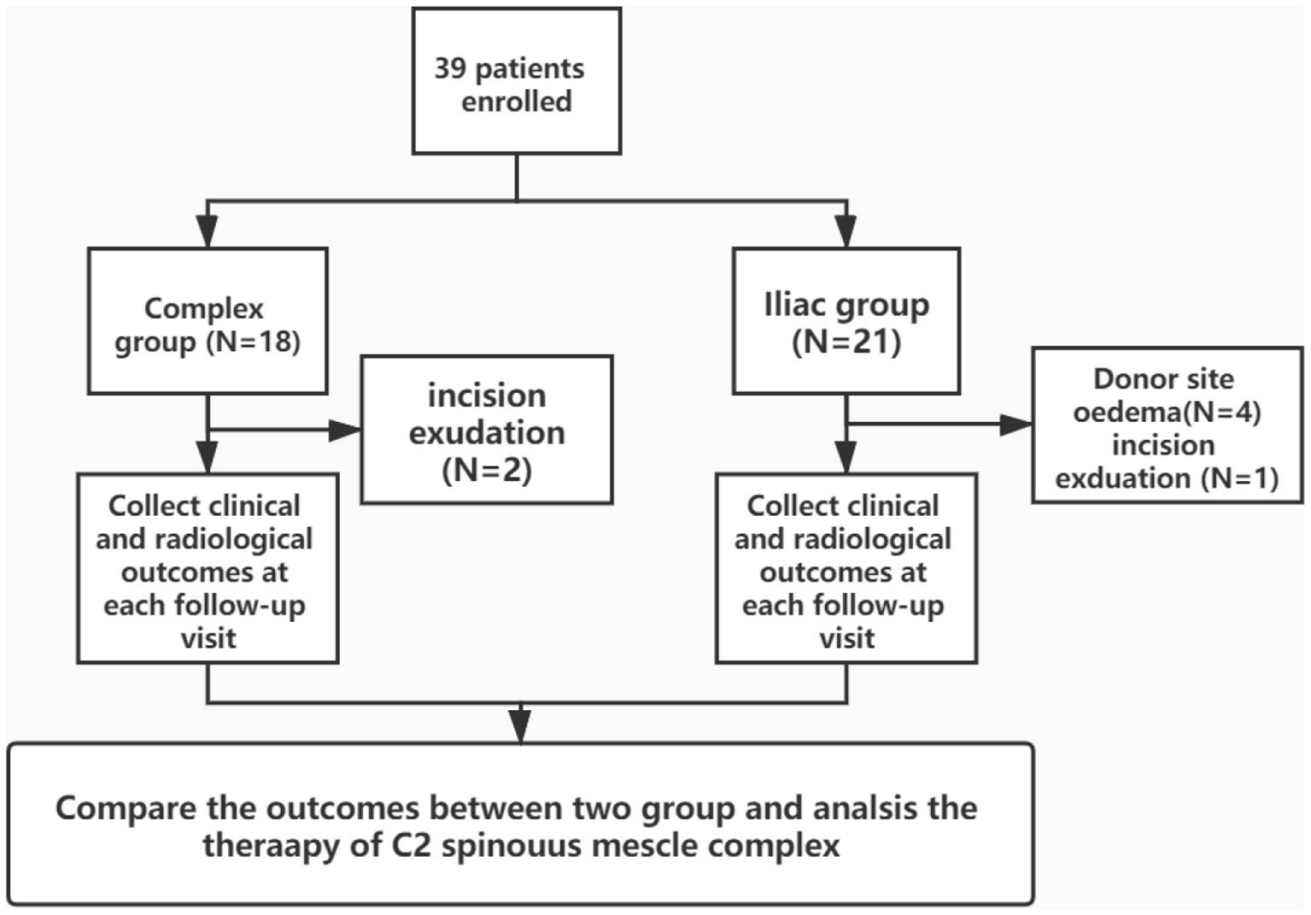
The diagram of study flows.

Inclusion criteria were as follows: (1) age >18 years; (2) presence of unstable Hangman fracture; and (3) absence of abnormal cervical vertebral abnormalities. Exclusion criteria were as follows: (1) presence of severe diseases such as cardiopathy; (2) presence of diseases that may influence bone structure, such as rheumatoid arthritis; and (3) absence of intact follow-up medical records. A total of 39 patients (complex group: 18 patients and iliac group: 21 patients) were enrolled in this retrospective comparative study (Figure 2). The clinical and demographic characteristics of the patients are presented in Table 1.

**Table 1:** The clinical and demographic characteristics of the two groups.

**Variable**	**Complex group**	**Iliac group**	*p*-value^*^
	**(*n* = 18)**	**(*n* = 21)**	
Mean age, years	49.1 ± 15.8	55.3 ± 12.2	0.16
**Gender**, ***n*** **(%)**
Male	12 (66.7%)	13 (61.9%)	0.51
Female	6 (33.3%)	8 (38.1%)	
**Fracture cause**, ***n*** **(%)**
Fall	5 (27.8%)	6 (28.6)	0.80
Vehicle injure	10 (55.5%)	13 (61.9%)	
Other	3 (16.7%)	2 (9.5%)	
**Fracture type**, ***n*** **(%)**^※^
II	11 (61.1%)	12 (57.1%)	0.85
IIA	4 (22.2%)	4 (19.1%)	
III	3 (16.7)	5 (23.8%)	
Mean time to surgery, days	3.3 ± 0.6	3.6 ± 0.7	0.29

* *Independent-samples t-test or Mann-Whitney U test for continuous variables; chi-squared test for categorical variables*.

※ *Levine and Edwards classification*.

Three patients in the complex group had associated injury, which included distal radial fracture (one patient) and proximal ulnar fracture (two patients). Similarly, patients in the iliac group also had associated injury, which included proximal humeral fractures (two patients). The associated injuries were treated conservatively.

### Outcome Evaluation

Clinical outcomes were measured using the visual analog scale (VAS) for pain (from 0 to 10, no pain to most severe pain) (10) and Japanese orthopedic association score (JOA) for cervical function, which consisted of four parts (from 0 to 17, higher score means better cervical function) (11). Radiological outcomes such as bone fusion and correct positioning were evaluated by cervical X-ray and CT-scan. Radiological assessments were performed by an experienced radiologist and a spine surgeon who worked together to establish consensus, but were not involved in the treatment or reporting of results.

### Surgical Technique

All patients were first treated with skull traction for 1–2 days with forces ranging from 3 to 5 kg for some degree of reduction.

### C2-3 Pedicle Screw Fixation With the C2 Spinous Muscle Complex

**Figure 3:**
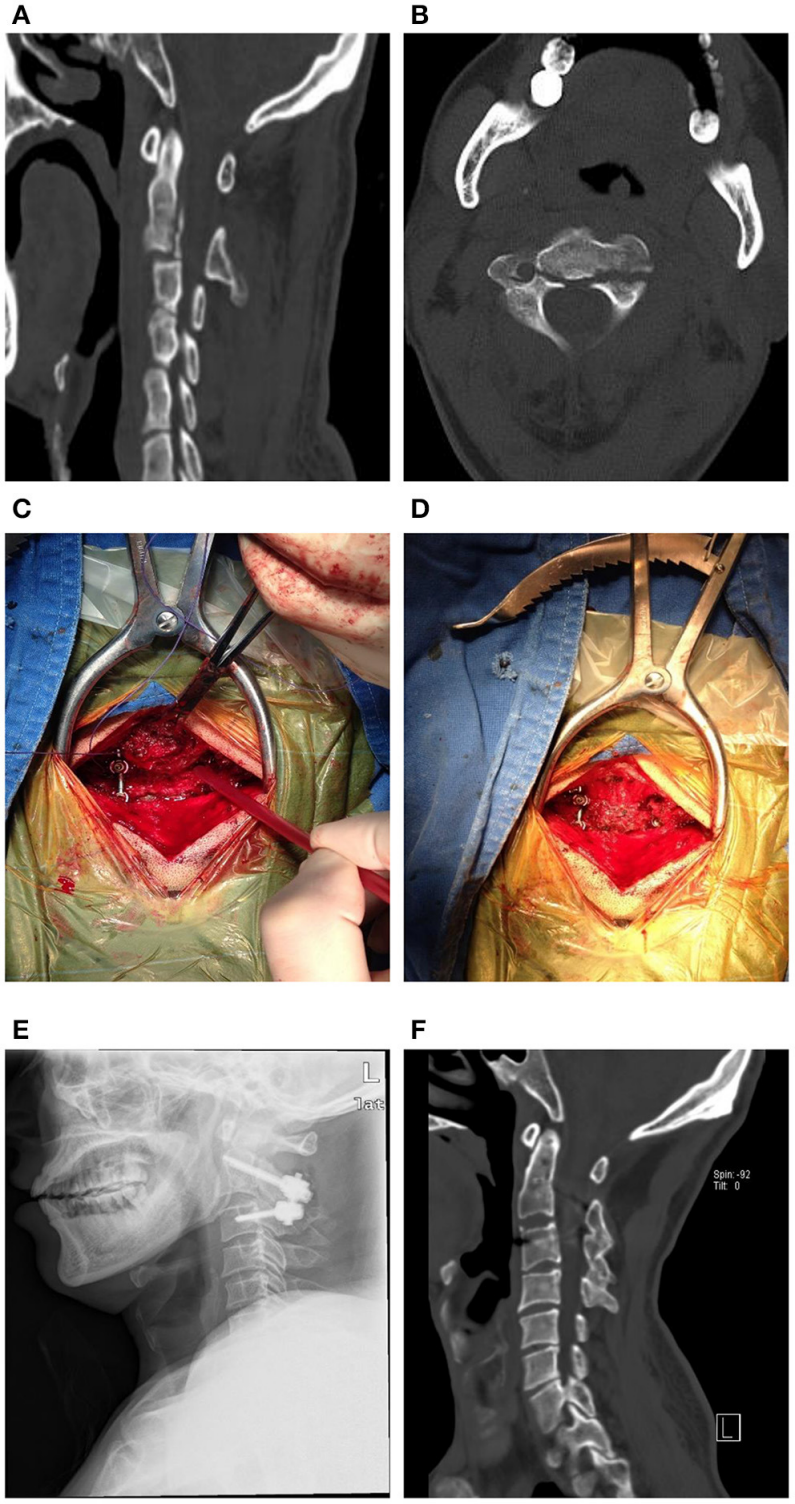
**(A,B)** Preoperative cervical CT-scan of a 42-year-old male patient with instable Hangman fracture. **(C,D)** The screws were inserted in C2-3 pedicle and C2 spinous muscle complex was displaced into C2-3 posterior arch. **(E)** The postoperative X-ray showed good reduction and realignment. **(F)** The posterior cervical CT-scan at 1-year follow-up visit showed the bone fusion.

The patients were positioned prone on the surgical table under general anesthesia, and their heads were fixed with a Mayfield frame (Integra LifeScience. Inc., American). Thereafter, a midline incision was made and the C2-3 posterior structures were exposed; however, the muscles were not detached from the C2 spinous process. The C2 pedicle screw (Shanghaisanyou, China) was inserted in the lateral mass of the C2, with a gantry angle of 15°-25° and an inward camber angle of 20°-25°. Subsequently, a C3 pedicle screw was inserted in the lateral mass of the C3 with a gantry angle of ~10° and an inward camber angle of 35°-45°. Thereafter, the posterior three-fourth of the C2 spinous was cut by a bone saw and displaced into the preprocessed C2-3 posterior arch. A rod was placed combined with pedicle screws to ensure tight contact between the C2 spinous muscle complex and the bone graft bed of the C2-3 posterior arch (Figure 3).

### C2-3 Pedicle Screw Fixation With the Iliac Bone Graft

The patients were positioned prone on the surgical table under general anesthesia, and their heads were fixed with a Mayfield frame (Integra LifeScience. Inc., Plainsboro, NJ, USA). Firstly, the iliac bones were obtained. Thereafter, a midline incision was made and the C2-3 posterior structures were exposed. The C2 pedicle screw (Shanghaisanyou, China) was inserted in the lateral mass of the C2, with a gantry angle of 15°-25° and an inward camber angle of 20°-25°. Subsequently, a C3 pedicle screw was inserted in the lateral mass of the C3, with a gantry angle of ~10° and an inward camber angle of 35°-45°. The bone graft bed was prepared in the C2-3 posterior arch and placed on the iliac bone. The rod was placed in combination with pedicle screws to ensure tight contact between the iliac bone and the bone graft bed.

The entire procedure was monitored under C-arm fluoroscopy. At the end of the surgery, CT scan was performed to confirm the fractural realignment and screw placement.

### Postoperative Protocol

The same postoperative protocol was applied in both the groups in this study. Patients were allowed out of bed on the second postoperative day with neck collar support, immobilized for at least 3 months, and kept away from smoking. After 2 weeks, the sutures were removed. The radiological outcomes and clinical outcomes were evaluated at each follow-up visit.

### Statistical Analysis

SPSS for Windows, Version 19.0, (SPSS Inc., Chicago, IL, USA) was used for all statistical analyses. Descriptive statistics are presented as mean ± standard deviation (SD). The means of parametric and non-parametric variables were compared between the two groups using the independent-samples *t*-test and the Mann–Whitney *U* test, respectively. The Chi-square test was used to compare categorical variables between the two groups. Statistical significance was set at *P* < 0.05.

## Results

In this study, all patients were followed up for >24 months, and no statistical difference was found in the clinical and demographic characteristics of the two groups (Table 1). Differences between preoperative and postoperative VAS and JOA scores were detected, without statistical significance (independent-samples *t*-test; *P* > 0.05). At 1-month follow-up visit, significant improvement in the VAS and JOA scores was found (independent-samples *t*-test; *P* < 0.05). Significant differences in the operative time, blood loss, and outcome measures were found between the two groups (Table 2). All patients had bone fusion at the final follow-up visit, and none of the patients had internal fixation failure.

**Table 2:** Comparison of surgical characteristics and outcome measures between two groups.

**Variable**	**Complex group (*n* = 18)**	**Iliac group (*n* = 21)**	*p*-value^*^
Blood loss, ml	224.4 ± 35.8	293.6 ± 45.6	<0.01^φ^
Operation time, h	2.2 ± 0.24	2.8 ± 0.32	<0.01^φ^
**Mean VAS**
Preoperative	5.5 ± 1.2	5.1 ± 0.8	0.48
1 month postoperative	2.5 ± 0.5	3.0 ± 0.7	0.012^φ^
6 months postoperative	1.1 ± 0.7	1.2 ± 0.6	0.21
12 months postoperative	0.8 ± 0.3	1.0 ± 0.4	0.12
24 months postoperative	0.5 ± 0.2	0.6 ± 0.3	0.52
**Mean JOA**
Preoperative	10.8 ± 1.0	10.5 ± 1.1	0.46
1 month postoperative	14.0 ± 0.7	13.1 ± 1.0	<0.01^φ^
6 months postoperative	15.2 ± 0.6	14.7 ± 0.8	0.34
12 months postoperative	16.1 ± 0.4	15.9 ± 0.6	0.62
24 months postoperative	16.4 ± 0.6	16.3 ± 0.3	0.35

* *Independent-samples t-test for parametric variables; Mann–Whitney U test for non-parametric variables*.

φ *P-Value < 0.05*.

*VAS, visual analog scale score; JOA, Japanese orthopedic association score*.

### Postoperative Complications

Complication data were obtained from the medical records. In the iliac group, four (19.0%) patients had superficial wound oedema in the donor site and one (4.8%) patient had exudation in the cervical midline incision, which was successfully managed by dressing exchange. Three (14.3%) patients who complained of donor-site pain and five (23.8%) patients who complained of neck pain were treated conservatively. In the complex group, two (11.1%) patients had exudation in the cervical midline incision, which was successfully managed by conservation treatment. A significant difference in postoperative complications was found between the two groups (Chi-square test, *P* < 0.05).

## Discussion

Instable Hangman fracture or traumatic spondylolisthesis of the axis is defined as the bilateral fracture of the axial pars interarticularis combined with severe circumferential disc and ligamentous injuries or a variable degree of translation or angulation of the C2 on the C3 vertebra (12). Most of the unstable hangman fractures are preferentially treated with surgery, including anterior graft and plating, C2 par fixation, C2-3 posterior pedicle screw fixation, and C2 lag screw-rod fixation (13–17). Recently, the most common surgical procedure that provides better biomechanical strength and has a short learning curve is C2-3 posterior pedicle screw fixation (18). Jeong et al. reported that the VAS and NDI scores of 13 patients with unstable Hangman fracture who underwent posterior C2-3 fixation had significantly improved after operation and were maintained up to the 12-month follow-up visit (final visit) (19). Similarly, Our group reported that 35 patients with unstable Hangman fracture who were treated with C2–C3 posterior short-segment fixation and fusion had satisfactory reduction and realignment without complications or reoperation (20).

However, some patients treated with C2-3 pedicle screw fixation and fusion may complain of neck pain and donor-site complications. Lang et al. reported that six patients with instable Hangman fracture who underwent minimally invasive C2-3 pedicle screw fixation showed better recovery in neck pain than those patients who were treated with conventional open surgery (21). Skeppholm et al. reported that 45 patients who underwent cervical decompression with bone graft showed donor-site complications at 1-year follow-up visit (22). Hence, we used C2-3 pedicle screw fixation with the C2 spinous muscle complex for instable Hangman fracture to minimize neck pain and donor-site complications.

In this study, the mean operative time was 2.2 h in the complex group and 2.8 h in the iliac group, and the mean blood loss was 224.4 mL in the complex group and 293.6 mL in the iliac group. The operative time and blood loss were significantly less in the complex group than in the iliac group (*P* < 0.01). Harvesting the autogenous iliac bone during surgery may result in longer operative time and greater blood loss.

The mean preoperative and 1-, 6-, 12-, and 24-months follow-up VAS scores were 5.5, 2.5, 1.1, 0.8, and 0.5, respectively, in the complex group and 5.1, 3.0, 1.2, 1.0, and 0.6, respectively, in the iliac group. A significant difference was found in the 1-month follow-up VAS scores of the two groups (*P* < 0.05). Similarly, a significant difference was found in the 1-month follow-up JOA scores of the two groups (*P* < 0.05). In addition, although differences in the JOA and VAS scores were detected at other visit times, they were non-significant (*P* > 0.05). Our group has reported that treatment with the C2 spinous muscle complex yields significantly superior outcomes with respect to operative time, blood loss, neck pain, and bone fusion time in patients with atlantoaxial instability (8). Lin et al. reported that 53 patients who were treated with open-door laminoplasty and unilateral preservation of the muscular-ligament complex had better cervical range of motion, C0-2 Cobb angle, and C2–C7 sagittal vertical axis than the 37 patients who underwent traditional open-door laminoplasty at the 16.7-month follow-up. Surgical damage to the posterior muscular-ligament complex may induce loss of cervical sagittal balance (23). Secer et al. reported that 27 patients with cervical spondylotic myelopathy underwent open-door laminoplasty with protection of muscles, and they yielded significant better results in neck ROM and cervical axial pain (24). Although, the axial spinous muscle complex preservation benefit in neck mobility postoperatively, Cheng et al. reported that 60 patients treated with cervical operation and reconstruction of C2 spinous process and muscle. Although the ROM in cervical spine has a decrease from 43.35° ± 7.55° to 34.83° ± 7.41°, but it has been significantly improved compared with the traditional method (25).

Armaghani et al. reported that of the 50 patients treated with anterior cervical discectomy and iliac bone graft fusion, two patients had superficial wound infection and a postoperative hematoma and five patients complained of donor-site pain (26). In addition, Silber et al. reported that of the 134 patients who underwent cervical surgery with iliac crest bone graft, 35 patients had donor-site pain and 21 patients had abnormal sensation at donor site (27). In our study, three (14.3%) patients had donor-site pain, four (19.0%) patients had incision oedema, and two patients had neck pain in the iliac group. In the complex group, no patient had neck pain; however, two (11.1%) patients had exudation in the cervical midline incision. A significant difference was found in neck pain and donor-site complications between the two groups (chi-square test, *P* < 0.05).

Therefore, it is not uncommon for patients complaining of donor-site complications. Consequently, the use of the C2 spinous muscle complex to displace the iliac bone not only prevents donor-site complications, but also improves cervical function and neck pain.

This study has some limitations. First, the sample size was small and the follow-up period was short, and we can enroll more patients for our further study. Second, we did not obtain patients who had undergone rehabilitation treatment or other life habits, such as smoking and physical work, which may have affected the clinical outcomes to some degree.

In conclusion, although C2-3 pedicle screw fixation with iliac bone graft is a widely used treatment for unstable Hangman fractures, patients may experience donor-site complications and neck pain. The use of the C2 spinous muscle complex, instead of the iliac bone, may yield better outcomes such as preventing donor-site complications, better recovery of neck pain and cervical function, reducing the operative time, and decreasing blood loss. However, more research is required to determine whether C2-3 pedicle screw fixation combined with C2 spinous muscle complex for unstable Hangman fractures can be performed prior to conventional C2-3 pedicle screw fixation with an iliac bone graft.

## Data Availability Statement

The raw data supporting the conclusions of this article will be made available by the authors, without undue reservation.

## Ethics Statement

The studies involving human participants were reviewed and approved by the Bioethics Committee of the Ningbo No.6 Hospital of Ningbo University. The patients/participants provided written informed consent to participate in this study.

## Author Contributions

DX, KG, YaW, YuW, and WM contributed to the study conception, design, and commented on previous versions of the manuscript. Material preparation, data collection, and analysis were performed by DX, KG, YaW, and WM. The first draft of the manuscript was written by DX. All authors read and approved the final manuscript.

## Funding

This study was conducted in accordance with the ethical principles of the Helsinki Declaration and was approved by the Ethics Committee of Ningbo Number 6 Hospital (approval number: 2019003), and the Nature Science Foundation of Zhejiang (LY19H060002).

## Conflict of Interest

The authors declare that the research was conducted in the absence of any commercial or financial relationships that could be construed as a potential conflict of interest.

## Publisher's Note

All claims expressed in this article are solely those of the authors and do not necessarily represent those of their affiliated organizations, or those of the publisher, the editors and the reviewers. Any product that may be evaluated in this article, or claim that may be made by its manufacturer, is not guaranteed or endorsed by the publisher.

## References

[1] Schneider RC Livingston KE Cave AJ Hamilton G. “Hangman's fracture” of the cervical spine. J Neurosurg. (1965) 22:141–54. 10.3171/jns.1965.22.2.0141 14288425

[2] Levine AM Edwards CC. The management of traumatic spondylolisthesis of the axis. J Bone Joint Surg Am Vol. (1985) 67:217–26. 10.2106/00004623-198567020-00007 3968113

[3] Salunke P Karthigeyan M Sahoo SK Prasad PK. Multiplanar realignment for unstable Hangman's fracture with Posterior C2-3 fusion: a prospective series. Clin Neurol Neurosurg. (2018) 169:133–8. 10.1016/j.clineuro.2018.03.024 29656174

[4] Duggal N Chamberlain RH Perez-Garza LE Espinoza-Larios A Sonntag VK Crawford NR. Hangman's fracture: a biomechanical comparison of stabilization techniques. Spine. (2007) 32:182–7. 10.1097/01.brs.0000251917.83529.0b 17224812

[5] Scheerlinck LM Muradin MS van der Bilt A Meijer GJ Koole R Van Cann EM. Donor site complications in bone grafting: comparison of iliac crest, calvarial, and mandibular ramus bone. Int J Oral Maxillofac Implants. (2013) 28:222–7. 10.11607/jomi.2603 23377069

[6] Sinha AK Goyal S. Myoarchitectonic advancement of the C2 spinous process for C1-C2 posterior fusion: a novel technique. J Neurosci Rural Pract. (2015) 6:267–71. 10.4103/0976-3147.153237 25883500 PMC4387831

[7] Ma W Zhao H Jiang W Xu N Hu X Li G. The effects of axial spinous process-muscle-vascellum complex transplantation for posterior atlantoarial fusion. Chin J Orthop. (2018) 15:927–34. 10.3760/cma.j.issn.0253-2352.2018.15.005

[8] Xu D Jiang W Ruan C Wang Y Hu X Chen Y. Efficacy comparison of posterior atlantoaxial screw-rod fixation combined with spinous process muscle-vessel complex bone graft or iliac bone graft for atlantoaxial instability. Chin J Trauma. (2019) 10:871–9.

[9] Riew KD Raich AL Dettori JR Heller JG. Neck pain following cervical laminoplasty: does preservation of the C2 muscle attachments and/or C7 matter? Evid Based Spine Care J. (2013) 4:42–53. 10.1055/s-0033-1341606 24436698 PMC3699245

[10] Dixon JS Bird HA. Reproducibility along a 10 cm vertical visual analogue scale. Ann Rheum Dis. (1981) 40:87–9. 10.1136/ard.40.1.87 7469530 PMC1000664

[11] Tetreault L Kopjar B Nouri A Arnold P Barbagallo G Bartels R. The modified Japanese orthopaedic association scale: establishing criteria for mild, moderate and severe impairment in patients with degenerative cervical myelopathy. Eur Spine J. (2017) 26:78–84. 10.1007/s00586-016-4660-8 27342612

[12] Effendi B Roy D Cornish B Dussault RG Laurin CA. Fractures of the ring of the axis. A classification based on the analysis of 131 cases. J Bone Joint Surg Br Vol. (1981) 63-b:319–27. 10.1302/0301-620X.63B3.7263741 7263741

[13] Wang S Wang Q Yang H Kang J Wang G Song Y. Novel technique for unstable Hangman's fracture: lag screw-rod (LSR) technique. Eur Spine J. (2017) 26:1284–90. 10.1007/s00586-016-4630-1 27246352

[14] Wang L Liu C Zhao Q Tian J. Posterior pedicle screw fixation for complex atlantoaxial fractures with atlanto-dental interval of >/= 5 mm or C2-C3 angulation of >/= 11 degrees. J Orthop Surg Res. (2014) 9:104. 10.1186/s13018-014-0104-5 25407360 PMC4245791

[15] Ge C Hao D He B Mi B. Anterior cervical discectomy and fusion versus posterior fixation and fusion of C2-3 for unstable hangman's fracture. J Spinal Disord Techn. (2015) 28:E61–66. 10.1097/BSD.0000000000000150 25099979

[16] Xu H Zhao J Yuan J Wang C. Anterior discectomy and fusion with internal fixation for unstable hangman's fracture. Int Orthop. (2010) 34:85–8. 10.1007/s00264-008-0658-0 18853157 PMC2899270

[17] Salunke P Sahoo SK Krishnan P Chaterjee D Sodhi HB. Are C2 pars-pedicle screws alone for type II Hangman's fracture overrated? Clin Neurol Neurosurg. (2016) 141:7–12. 10.1016/j.clineuro.2015.11.019 26716722

[18] Chittiboina P Wylen E Ogden A Mukherjee DP Vannemreddy P Nanda A. Traumatic spondylolisthesis of the axis: a biomechanical comparison of clinically relevant anterior and posterior fusion techniques. J Neurosurg Spine. (2009) 11:379–87. 10.3171/2009.4.SPINE08516 19929332

[19] Jeong DH You NK Lee CK. Posterior C2-C3 fixation for unstable Hangman's fracture. Korean J Spine. (2013) 10:165–9. 10.14245/kjs.2013.10.3.165 24757480 PMC3941766

[20] Ma W Xu R Liu J Cho KH Kim SH. Posterior short-segment fixation and fusion in unstable Hangman's fractures. Spine. (2011) 36:529–33. 10.1097/BRS.0b013e3181d60067 21079544

[21] Lang Z Tian W Liu Y Liu B Yuan Q Sun Y. Minimally invasive pedicle screw fixation using intraoperative 3-dimensional fluoroscopy-based navigation (CAMISS Technique) for hangman fracture. Spine. (2016) 41:39–45. 10.1097/BRS.0000000000001111 26267827

[22] Skeppholm M Olerud C. Pain from donor site after anterior cervical fusion with bone graft: a prospective randomized study with 12 months of follow-up. Eur Spine J. (2013) 22:142–7. 10.1007/s00586-012-2456-z 22890567 PMC3540321

[23] Lin S Zhou F Sun Y Chen Z Zhang F Pan S. The severity of operative invasion to the posterior muscular-ligament complex influences cervical sagittal balance after open-door laminoplasty. Eur Spine J. (2015) 24:127–35. 10.1007/s00586-014-3605-3 25307698

[24] Secer HI Harman F Aytar MH Kahraman S. Open-door laminoplasty with preservation of muscle attachments of C2 and C7 for cervical spondylotic myelopathy: retrospective study. Turk Neurosurg. (2018) 28:257–62. 10.5137/1019-5149.JTN.20007-17.1 28345126

[25] Cheng Z Chen W Yan S Li W Qian S. Expansive open-door cervical laminoplasty: *in situ* reconstruction of extensor muscle insertion on the c2 spinous process combined with titanium miniplates internal fixation. Medicine (Baltimore). (2015) 94:e1171. 10.1097/MD.0000000000001171 26181563 PMC4617069

[26] Armaghani SJ Even JL Zern EK Braly BA Kang JD Devin CJ. The evaluation of donor site pain after harvest of tricortical anterior iliac crest bone graft for spinal surgery: a prospective study. Spine. (2016) 41:E191–196. 10.1097/BRS.0000000000001201 26571154

[27] Silber JS Anderson DG Daffner SD Brislin BT Leland JM Hilibrand AS. Donor site morbidity after anterior iliac crest bone harvest for single-level anterior cervical discectomy and fusion. Spine. (2003) 28:134–9. 10.1097/00007632-200301150-00008 12544929

